# Early nomads of the Eastern Steppe and their tentative connections in the West

**DOI:** 10.1017/ehs.2020.18

**Published:** 2020-05-07

**Authors:** Alexander Savelyev, Choongwon Jeong

**Affiliations:** 1 Max Planck Institute for the Science of Human History, 07745 Jena, Germany; Institute of Linguistics, Russian Academy of Sciences, Bolshoy Kislovsky pereulok 1/1, 125009 Moscow, Russia; 2 School of Biological Sciences, Seoul National University, Gwanak-gu, 08826 Seoul, Republic of Korea

**Keywords:** Xiongnu, Rourans, Huns, Avars, history of the Steppe

## Abstract

The origin of the Xiongnu and the Rourans, the nomadic groups that dominated the eastern Eurasian steppe in the late first millennium BC/early first millennium AD, is one of the most controversial topics in the early history of Inner Asia. As debatable is the evidence linking these two groups with the steppe nomads of early medieval Europe, i.e. the Huns and the Avars, respectively. In this paper, we address the problems of Xiongnu–Hun and Rouran–Avar connections from an interdisciplinary perspective, complementing current archaeological and historical research with a critical analysis of the available evidence from historical linguistics and population genetics. Both lines of research suggest a mixed origin of the Xiongnu population, consisting of eastern and western Eurasian substrata, and emphasize the lack of unambiguous evidence for a continuity between the Xiongnu and the European Huns. In parallel, both disciplines suggest that at least some of the European Avars were of Eastern Asian ancestry, but neither linguistic nor genetic evidence provides sufficient support for a specific connection between the Avars and the Asian Rourans.

## Graphical abstract

**Figure:**
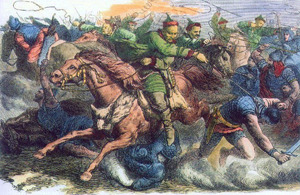

**Media summary**: Historical linguistics and population genetics provide insights into the Xiongnu–Hun and Rouran–Avar tentative connections.

## 1. Introduction

Since very early in history, Inner Asia – i.e. Mongolia and the adjacent parts of southern Siberia and northern China – has been a home for diverse ethnic groups and a place where distinct cultural, linguistic and genetic lineages have come together and interacted in multiple ways. There is good reason to believe that the ethnic map of the region in prehistory was, in some respects, even more complex than it is today, because some of its early inhabitants became extinct afterwards, and some left their homeland to settle across the vast expanse of northern Eurasia (see, e.g. Janhunen, 1996, 2010; Shimunek, 2017; Robbeets et al., in press, for different accounts of this issue).

One of the most intriguing topics in the early history of Inner Asia is the genesis and the destiny of the early nomadic groups that, as witnessed by the contemporary Chinese sources, populated the areas north of China approximately from 300 BC to 550 AD. Those include, among others, the Xiongnu (Hsiung-nu) of the third century BC to the second century AD and the Rourans (Ruan-ruan, Jou-jan) of the fourth to sixth centuries AD. Both groups disappeared from Chinese sources soon after the collapse of the Xiongnu and, subsequently, Rouran steppe empire. An oft-cited view is, however, that the Xiongnu and, several centuries later, the Rourans were not entirely assimilated by the neighbouring peoples. Some of them may have migrated westwards across the Eurasian steppe zone and become ancestors to the first settlers of Inner Asian origin in Europe of the Great Wandering period, i.e. the European Huns and the European Avars, respectively (Golden, 1993).

This traditional interpretation was contested in more recent studies, although to a different extent. While claims about the Xiongnu/Hunnic connection are now often seen as uncertain or even obviously wrong, the assumed continuity between the Rourans and the Avars is much more widely accepted (e.g. Róna-Tas and Berta, 2011). Overall, there are still many gaps in our understanding of the population movements, language dispersals and cultural dynamics in the steppe at that time. This is largely due to the scarcity and ambiguity of the data available to individual disciplines focused on the human past, be it written history, archaeology, historical linguistics or population genetics. Further progress in the field seems to depend crucially on interdisciplinary research, involving all of these disciplines.

In this article, we adopt such an interdisciplinary approach in order to infer the cultural, linguistic and genetic origins of the early nomads of the Eastern Steppe as well as their tentative descendants in the West. We restrict ourselves to two case studies. The first study (Section 2) focuses on the Xiongnu of Chinese sources and the Huns of Europe, and the second study (Section 3) examines the origins of the Rourans and the Avars. What these case studies have in common is, first, the almost identical geographical setting. In a similar way as the Rourans ‘restored’ the empire of the Xiongnu in Inner Asia in the fourth century AD, the Avars of the sixth century founded their own empire on what had previously been the core Hun territory in Central Europe. The second point in common is the controversy about the extent of (dis)continuity, a key problem in both Xiongnu/Hunnic and Rouran/Avar studies. Third, what is a specific point of interest for us is the oft-discussed association of all or some of the groups in question with the Altaic world. Previously, for each of these groups a linguistic identification with one of the Altaic branches has been proposed (most often Turkic, or sometimes Mongolic, or Tungusic occasionally). An evaluation of these hypotheses against the background of known ‘non-Altaic’ proposals is another major objective of this paper.

In what follows below, we first present overviews of written historical accounts and the state of the art in the archaeology of the discussed steppe nomadic groups. Then we provide a critical evaluation of the available linguistic evidence and, finally, report on recent progress in population genetics of the steppe. It must be noted that each line of evidence is examined independently from what is known in the other disciplines, so that we avoid circular argumentation. Thereafter, in Section 4, we merge the evidence drawn from individual disciplines in order to provide insights into the Xiongnu/Hunnic and Rouran/Avar problems within an interdisciplinary context.

## 2. The Xiongnu of Inner Asia and the Huns of European history

### 2.1. Historical and archaeological background

**Figure 1.**
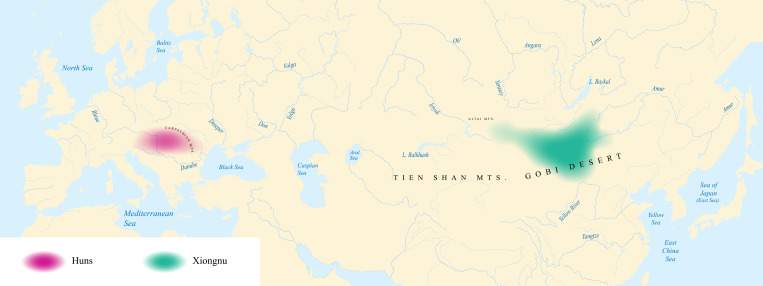
The Xiongnu steppe empire and the heartland of the European Huns.

The Xiongnu were a steppe people who dominated the areas north of China between the third century BC and the second century AD (Figure 1). The only written accounts of the Xiongnu history were left by their major rivals in the region, the Han Chinese. The contemporary Chinese authors considered the Xiongnu under the general label of the ‘Northern Barbarians’ (胡 Hu). Between 209 and 161 BC, under the rule of Maodun and his son Jiyu, the Xiongnu brought under their control neighbouring tribes and established a powerful confederation encompassing much of Mongolia, China's Inner Mongolia and Southern Siberia (Golden, 1993: 61). The ‘super-complex chiefdom’ of the Xiongnu, to use a term by Kradin (2011: 93), was a multiethnic steppe empire, the first documented polity of that kind in Inner Asia (Brosseder and Miller, 2011). The Xiongnu economy was dependent on nomadic pastoralism, although it had an agricultural component as well (Savelyev, 2017: 127–128).

Skilled in weaponry and warfare, the Xiongnu waged constant wars with China for supremacy in the Ordos region, leading to mixed results. Owing to increasing Chinese dominance and internal tensions, the Xiongnu empire started to decline in the first century BC. Around 49 AD the Xiongnu had to divide their realm into two parts. The polity of the Southern Xiongnu was subjugated by the Han dynasty. It was used by the latter as a buffer state between China and the Northern Xiongnu empire, which remained independent for around a century. The Northern Xiongnu were defeated by the Mongolic-speaking Xianbei in 155 AD.

The archaeological record of the Xiongnu is relatively well known, with the caveat that identification of what has been seen as the Xiongnu archaeological culture with the Xiongnu of historical sources requires some caution. As noted by Di Cosmo (2011: 36–37), ‘no historical event or historical representation of the Xiongnu other than in very rare cases can be firmly connected with an actual Xiongnu archaeological culture or population’. Nor is there consensus on what region was the ancestral homeland of the Xiongnu. The Ordos Plateau in Inner Mongolia, Manchuria and Southern Siberia is discussed in the literature as possible areas of the Xiongnu *Urheimat*; alternatively, the descendance of the Xiongnu from the Slab Grave culture population, who preceded them in eastern Mongolia, is debated (Lee and Linhu, 2011). The ethnic origins of the core Xiongnu population are as much an unresolved question, from an archaeological viewpoint. It is still likely that the Xiongnu included an Eastern Iranian (Saka) component or were at least strongly influenced by the Iranians. It is also arguable that the Xiongnu learned the steppe nomadic model of economy from their Eastern Iranian neighbours (Beckwith, 2009: 72–73, 404).

The relationship between the Xiongnu of Inner Asia and the Huns who became known to European sources during the Great Wandering of Peoples, is a great controversy. Once widely accepted, the theory that the groups were indeed related has only limited support in modern scholarship. The names of the Xiongnu and the Huns may be of the same origin (cf. Atwood, 2012), but this does not assure any sort of political, cultural, linguistic or ethnic continuity. Beckwith (2009: 72) emphasizes the lack of any known direct connection between the two steppe populations. On the other hand, La Vaissière (2005a, b, 2014) has recently given a fresh impetus to the idea that there had been a sort of political and, to some extent, cultural continuity between the Xiongnu and the Huns, based on Chinese and Sogdian written sources. From an archaeological perspective, the proper ‘Hunnic’ components in the archeological record of the European Huns have few parallels in regions of the Xiongnu dominance in Asia (Pohl, 2019: 105). Overall, the archaeological steppe heritage among the European Huns is very limited (Schmauder, 2009).

The Huns arrived on the doorstep of Europe in the 370s. They defeated the Alans and the Ostrogoths in the Pontic Steppe area and headed towards Roman territory, subduing other ‘Barbarian’ peoples on the way. In the 390s, the Huns occupied the Carpathian Basin, which became the power base of the emerging confederation (Figure 1). The Hun steppe empire reached the zenith of expansion under the rule of Attila in the mid-fifth century and suffered a rapid decline after his death in 453. New steppe polities came on the scene in different parts of what had previously been Attila's realm. Showing different degrees of continuity with the Hun empire, most of them were no less short-lived.

### 2.2. Historical linguistics

The problem of the affiliation of the Xiongnu/Hunnic language(s) is a long-standing controversy in historical linguistics. Owing to the scarcity of unequivocal evidence, some scholars (e.g. Doerfer, 1973) even considered this/these language(s) unclassifiable – a position that was reproduced recently by Shimunek (2017). The traditional and prevailing view is, however, that the Xiongnu and/or the Huns were Turkic (Shiratori, 1900; Benzing, 1959; Tenišev, 1997; Schönig, 1997–1998; Dybo, 2007; Janhunen, 2010) or, at least, Altaic speakers (Pritsak, 1982). Alternative hypotheses include their identification with the speakers of Eastern Iranian (Bailey, 1985: 25) or groups of ‘Paleo-Asiatic’, namely Yeniseian, origin (Vovin, 2000, 2002; Vovin et al., 2016, an idea going back to Ligeti, 1950, and Pulleyblank, 1962).

Most attempts at identifying the language of the Xiongnu of Inner Asia revolve around the only attested text that can be associated with this language, the so-called Jie couplet. Recorded with Chinese characters, it was included in Jin Shu, a history of the Jin dynasty period. The chronicle itself was composed in the mid-seventh century AD, but the couplet is given in a context referring to what happened in 307–311 AD in the lands of the Jie ‘Barbarians’, who were considered a branch of the Xiongnu. While a Chinese translation for the couplet is provided in Jin Shu, there are some major obstacles to its reliable interpretation. Those include the brevity of the couplet, which consists of only four words, and the debated status of the reconstruction of Old Chinese phonology, which is required for an adequate reading of the text (see Karlgren, 1954; Pulleyblank, 1962; Starostin, 1989; Baxter and Sagart, 2011, for its most authoritative versions). Problematic issues in the reconstructions of the known protolanguages that are compared with the Xiongnu linguistic data are also part of the question.

More than a dozen readings of the Jie couplet are available in the literature, and most of them identify its language as an early Turkic variety. Those include the oft-cited reading by Ramstedt (1922), who based himself on Shiratori (1900) and was followed by Bazin (1948) and Gabain (1949). The latter two readings had their advantages but were not a real breakthrough against the background of that by Ramstedt. A major advance was the reading by Shervashidze (1986), who used more up-to-date versions of both Old Chinese and Proto-Turkic phonological reconstructions. His interpretation was further elaborated by Dybo (2007), who identified the Jie language with Late Proto-Turkic. Recently, Shimunek et al. (2015) have offered yet another Turkic-based reading of the couplet. However, first, they argue against the Xiongnu affiliation of the Jie ‘Barbarians’ for historical reasons. Second, this reading is hardly compatible with the current state of the Turkic historical phonology; see Vovin et al. (2016) for a partly fair criticism.

An identification of Jie as a closest relative of the Yeniseian Pumpokol language was proposed in Vovin (2000) and further explored in Vovin (2002) and Vovin et al. (2016). For several reasons, this interpretation is unfortunate, as compared with the Turkic-based readings. Of the four words of the couplet, only two may, very tentatively, be read based on Pumpokol data; and both include lexical and grammatical morphemes that are not actually attested in Pumpokol. Regarding the other two words, which do not have even hypothetical parallels in Yeniseian, Vovin et al. (2016) have to assume a loan from an unidentified language into the Pumpokolic language of the Xiongnu. This makes the whole reading quite doubtful.

In addition to the Jie couplet, Chinese sources contain numerous glosses for isolated Xiongnu words, which were collected by Pulleyblank (1962). Most of them are, however, personal names that allow for a very broad range of interpretations. Therefore, it is only several dozens of words that are usually discussed in works on the affiliation of the Xiongnu language.

Pulleyblank (1962) attempted to connect the Xiongnu glosses with known Yeniseian lexemes. Some of his Xiongnu–Yeniseian etymologies are generally unconvincing, and some – the more plausible ones – may be part of shared cultural vocabulary, of non-native origin, in both Xiongnu and Yeniseian. A recent and more up-to-date interpretation of the 56 most transparent Xiongnu etymologies was provided by Dybo (2007). According to her analysis, the lexicon associated with the Xiongnu of written history is partly Eastern Iranian and partly Turkic in origin. The two lexical strata are differently distributed as regards their chronology and semantics. The words of Eastern Iranian origin occur in the earlier, mostly Western Han, sources and are almost entirely titles and terms for dairy products, in addition to the word for ‘comb’. The Turkic words occur both in early and late sources, and across various semantic domains. In addition, isolated Tocharian and Mongolic forms may have been present here, but these etymologies are the least transparent part of the Xiongnu vocabulary. The results of Dybo's analysis of the Xiongnu lexical material are summarized in Table 1.

**Table 1.** The distribution of Xiongnu glosses according to their origin (adapted from Dybo, 2007)

	Common nouns (more reliable etymologies)		Proper names, titles, ethnonyms (less reliable etymologies)	
	Early period	Late period	Early period	Late period
Proto-Turkic	11	8	7	3
Eastern Iranian	8	1	15	
Tokharian	1		1	
Mongolic	1			

Such a distribution of Xiongnu words may be an indication that both Turkic and Eastern Iranian-speaking groups were present among the Xiongnu in the earlier period of their history. Etymological analysis shows that some crucial components in the Xiongnu political, economic and cultural package, including dairy pastoralism and elements of state organization, may have been imported by the Eastern Iranians. Arguably, these Iranian-speaking groups were assimilated over time by the predominant Turkic-speaking part of the Xiongnu population.

The language of the European Huns is sometimes referred to as a Bulghar Turkic variety in general linguistic literature, but caution is needed in establishing its affiliations. The remnants of the Hunnic language are limited and ambiguous in interpretation: it is mostly personal names with only a few common nouns, including titles. Priscus witnessed that two ‘Barbarian’ languages were spoken in the camp of Attila, i.e. Gothic and Hunnic. At least some of the names of the Huns must have been Gothic in origin, including the names of Attila (Gothic *atta* ‘father’ + the diminutive suffix *-ila*) and his relatives as well as high officials. Some persons referred to as Huns in the sources from the post-Attila period bore names of apparently Alanic (Ossetic) origin, e.g. *Αψίχ* [*Apsikh*] ‘Hun officer in the Byzantine army about 540’, cf. Digor Ossetic *æfsæ* ‘mare’ < PIr. *aspa- ‘horse’ (Maenchen-Helfen, 1973: 422). According to Maenchen-Helfen's estimate, most of the Hunnic names are of Turkic origin, e.g. *Mundzuc* ‘the name of Attila's father’, cf. PTk *bunčuq ‘war flag, standard’ > *munčuq* in most Turkic subgroups except Oghuz. However, the majority of the previously proposed Turkic etymologies for the Hunnic names are far from unambiguous, so no firm conclusion can be drawn from this type of data.

The Hunnic titles are common titles of the nomadic steppe world. Most of them are attested in Turkic, but their ultimate origins may lie outside the Turkic family, as is most likely the case for the title of khagan (*χαγάνος*, *chaganus*) < ? Middle Iranian *hva-kama- ‘self-ruler, emperor’ (Dybo, 2007: 119–120). The few non-titles in the Hunnic common noun vocabulary are all of local (Indo-European) origin: *κάμος* [*kamos*] ‘a drink of barley’, *μέδος* [*medos*] ‘an alcoholic drink’, *strava* ‘lamentation’ (Maenchen-Helfen, 1973: 424–427).

### 2.3. Population genetics

The ancestry and genetic diversity of people who constituted the Xiongnu confederation have been of great interest by human geneticists. Most genetic studies on ancient Xiongnu people focused on uniparental markers (mitochondrial DNA and Y chromosome). These studies found a mixture of haplogroups from western and eastern Eurasian origins that suggested a large genetic diversity within, and possibly multiple origins of, Xiongnu elites (Kim et al., 2010; Pilipenko et al., 2018). Recently, genome-wide data of a few Xiongnu individuals, as well as temporally preceding populations in nearby regions (e.g. southern Russia and Kazakhstan), have been published, providing the first look into the genomic profile of Xiongnu (Damgaard et al., 2018a; Unterländer et al., 2017; Allentoft et al., 2015).

Although counting to only three, Xiongnu genomes from Mongolia are genetically diverse and show signatures of east-west admixture: one is mostly of eastern Eurasian origin while the other two are clearly admixed between western and eastern Eurasian sources, much like earlier Iron Age genomes from nearby regions, including southern Russia, Kazakh steppe and the Tian Shan mountains. Their genetic profiles are distinct from the Late Bronze Age individuals from either northern Mongolia or southern Siberia, which can be modelled as a mixture of a western source related to Andronovo/Sintashta/Srubnaya culture and an eastern one associated with hunter–gatherers from the Baikal region (Jeong et al., 2018; Allentoft et al., 2015; Damgaard et al., 2018b). Specifically, individuals from Iron Age steppe and Xiongnu have an ancestry related to present-day and ancient Iranian/Caucasus/Turan populations in addition to the ancestry components derived from the Late Bronze Age populations. We estimate that they derive between 5 and 25% of their ancestry from this new source, with 18% for Xiongnu (Table 2). We speculate that the introduction of this new western Eurasian ancestry may be linked to the Iranian elements in the Xiongnu linguistic material, while the Turkic-related component may be brought by their eastern Eurasian genetic substratum.

**Table 2.** Admixture modelling of Iron Age steppe groups and Xiongnu

Target	*P*-value	P-value for the third source	Sintashta MLBA	Khovsgol LBA	Gonur1 BA
IA steppe	0.144	0.059	0.358 (0.026)	0.592 (0.011)	0.050 (0.026)
Russia IA	0.845	8.61 × 10^−9^	0.261 (0.028)	0.573 (0.011)	0.166 (0.028)
Sarmatian	0.106	<10^−15^	0.667 (0.018)	0.141 (0.007)	0.192 (0.018)
Saka (Kazakhstan)	0.231	4.11 × 10^−3^	0.399 (0.026)	0.524 (0.011)	0.077 (0.027)
Saka (Tian Shan)	0.625	<10^−15^	0.483 (0.024)	0.269 (0.009)	0.248 (0.025)
Xiongnu	0.111	6.16 × 10^−6^	0.239 (0.038)	0.582 (0.016)	0.178 (0.040)

All six target groups are adequately modelled as a three-way mixture of Bronze Age western steppe (Sintashta MLBA), Late Bronze Age Mongolian (Khovsgol LBA) and an Iranian-related source from Bronze Age Uzbekistan (Gonur1 BA). The Iranian-related ancestry is significantly bigger than zero for all but one case (*p* << 0.05), which has a marginal *p*-value (0.059). Numbers in parentheses represent standard error estimates. Genomic data are obtained from the following publications: IA steppe (Unterländer et al., 2017), Russia IA (Damgaard et al., 2018a), Sarmatian (Damgaard et al., 2018a; Mathieson et al., 2015; Unterländer et al., 2017; Krzewińska et al., 2018), Saka and Xiongnu (Damgaard et al., 2018a), Sintashta and Gonur1 (Narasimhan et al., 2019) and Khovsgol (Jeong et al., 2018).

The genetic profile of published Xiongnu individuals speaks against the Yeniseian hypothesis, assuming that modern Yeniseian speakers (i.e. Kets) are representative of the ancestry components in the historical Yeniseian speaking groups in southern Siberia. In contrast to the Iron Age populations listed in Table 2, Kets do not have the Iranian-related ancestry component but harbour a strong genetic affinity with Samoyedic-speaking neighbours, such as Selkups (Jeong et al., 2018, 2019).

## 3. The Rourans of Inner Asia and the Avars of European history

### 3.1. Historical and archaeological background

**Figure 2.**
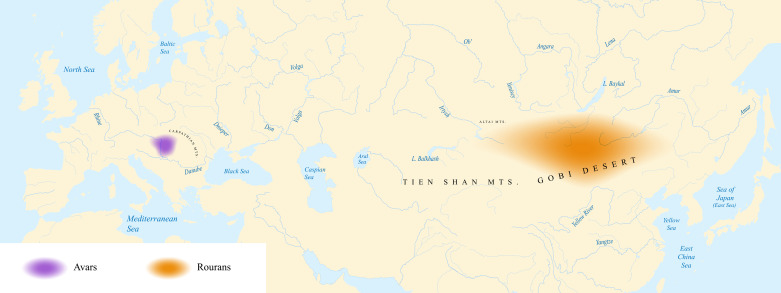
The Rouran steppe empire and the heartland of the European Avars.

The Rourans ruled Mongolia and adjacent areas, stretching from the Tian Shan to the Altai mountains, between the mid-fourth and mid-sixth centuries AD (Figure 2). Their empire occupied approximately the same territory that had previously been dominated by the Xiongnu and then by the Xianbei. The extent of continuity between the Rourans and their predecessors is, however, not fully established (Golden, 1993: 77). The Rouran confederation gained power during the second half of the fourth century AD and took control over most of Inner and Central Asia in the fifth century. As much as other steppe empires of the early medieval period, the population of the Rouran empire must have included components of diverse ethnic backgrounds.

The Rourans were defeated by the Turks, who had been their subjects, in 552–555. Their empire fell apart and, according to the contemporary Chinese sources, the core Rouran population was brutally massacred. Some of the Rourans fled to China and soon disappeared from sources. Another group of the Rourans is commonly thought to have migrated westwards and become the Avars of European history (e.g. Róna-Tas and Berta, 2011: 24).

The question of identification of the Inner Asian Rourans and the European Avars is in fact very complex and, as shown by Pohl (2019), it cannot be approached in a straightforward manner. Based on the interdisciplinary evidence that is discussed below, the Avars must have included a component originating in the Eastern steppes, but it cannot be safely identified specifically with the Rouran elites. In his account of this controversy, Pohl (2019: 38–47) resurrects the old hypothesis according to which the European Avars were actually ‘Pseudo-Avars’, a steppe group of mixed origin whose elite adopted the prestigious name of the ‘true’ Avars/Rourans for the purpose of political legitimization.

While there is almost no archaeological evidence available from the Rouran empire (Kradin, 2005: 150), the rich archaeological record of the European Avars supports their diverse origins. In the early period, there was no homogeneous ‘Avar archaeological culture’ in the Avar realm. Instead, heterogeneous components of eastern steppe origin coexisted with those having striking parallels in the Caucasus, the Southern Russian steppes and West Asia, also showing close links to the local European traditions, Mediterranean and Merovingian in the first place (Vida 2005: 17). Objects and customs from the Central Asian steppe do not even constitute the bulk of the early Avar-period material, according to Pohl (2019: 101).

The Avars reached Eastern Europe in the 550s and stayed in the Northern Caucasus region for several years. In the winter of 557/558, they contacted for the first time the Byzantines through the Alans. In 563, the Avars were already on the Danube. In 567, they entered the Carpathian basin and defeated the Germanic tribe of Gepids, and a year later they occupied the adjacent Lombard lands. On this territory, where the Roman Pannonia and the metropole of Attila's Huns were once located, they founded the Avar Khaganate (Figure 2). By the 580s, this steppe polity had gained enough military power to become the major rival of the Byzantine empire in the north.

In the early seventh century, the Avars brought under their control much of the Central and Eastern European steppe area. Remarkably, at this very time, i.e. after ca. 600 AD, the Avar archaeological record in the Carpathian basin becomes much more homogeneous as compared with the previous period. This homogenization was further enhanced in the remaining 200 years of Avar rule in the region.

During the seventh and eighth centuries, the Avars were involved in numerous wars and internal conflicts. The Avar Khaganate was defeated by Charlemagne's Franks in 796, and the Avars, as a group, disappeared completely from the map of Europe just for 20 years. The survivors from Charlemagne's military campaign were rapidly Slavicized, perhaps well before the Hungarian conquest of the Carpathian Basin in 895.

### 3.2. Historical linguistics

There is almost no direct evidence on the ‘Rouran’ language, if it ever existed as a separate linguistic entity. Therefore, most proposals on the linguistic affiliation of the Rourans start from rather speculative assumptions. Quite often, these are based on arguments that are non-linguistic in nature, such as Boodberg's (1935) account of Rouran as a para-Mongolic language.

In his two papers, Vovin (2004, 2010) proposed to identify Rouran as a source of substratum loans of unclear origin in Old Turkic. Some of the words in question are also attested in other Common Turkic languages but none, allegedly, in the Bulghar Turkic branch. However, for most of these lexemes, a substratum origin has never been properly demonstrated. Some terms on Vovin's list are not actually isolated in Common Turkic since they have Bulghar Turkic cognates (e.g. Old Turkic *küskü* ‘rat’ ~ the Danube Bulghar source of Hungarian *güzü* ‘gleaner mouse’; Róna-Tas and Berta, 2011: 361–362). Some other words on the list do have reliable etymologies within Turkic and are, thus, not of ‘unknown origin’ (e.g. Old Turkic *alp* in *alp-aɣïr* ‘difficult, hard’ is identical to *alp* ‘giant’, lit. ‘giant-difficult’ = ‘enormously difficult’; Old Turkic *alqu* ‘all’ is based on *al-* ‘to take’, see Erdal, 2004: 225–226; Dybo, 2013: 53).

Quite recently, Vovin (2019a, b) has adopted La Vaissière's (2018) alternative proposal, identifying Rouran with the ‘earliest Mongolic language’ of the newly read Brāhmī Bugut, Khüis Tolgoi and Keregentas inscriptions in Brāhmī script from central Mongolia and eastern Kazakhstan (see also Vovin, 2018). The Mongolic interpretation of the two more readable inscriptions, the Brāhmī Bugut and the Khüis Tolgoi, is quite convincing, but their identification with the Rouran language remains problematic because of the dating. According to Vovin (2019a), the Brāhmī Bugut inscription is dated to ca. 584–587 AD, and the Khüis Tolgoi inscription must have been erected between 604 and 620 AD. As both were created several decades after the Rouran Khaganate had been destroyed, it is unsafe to make conclusions on the composition of the Rouran population, or its elite, on the basis of these inscriptions. Yet one cannot exclude that some groups among the Rourans did speek a Mongolic language (e.g. note the close historical connection of the Rourans with the Mongolic-speaking Xianbei).

It was Doerfer (1967: 136) who proposed a Rouran origin for several titles that do not have reliable etymologies in known languages and were, arguably, first attested among the Rourans, such as *χan* ‘king, khan’, *χaγan* ‘khagan, great khan’, *xatun* ‘khan's wife’. These titles were later used by the Hunnic peoples and the European Avars, but also by other groups of different linguistic background in the steppe and beyond. The ultimate source of these words is subject to discussion. Vovin's (2010) attempt to interpret the titles *χan* and *χaγan* as Yeniseian roots (*qɛ ‘great, big’, *qʌj ‘ruler’) inflected with a Tabγač nominal suffix *-n* is quite unreliable: one could explain any word in any language if unattested hybrid formations of this kind were seen as a decent etymological solution. Following Benveniste (1966), Dybo (2007: 106–107) considers Turkic *χatun ‘king's wife’ a word of ultimate Eastern Iranian origin, borrowed presumably from Early Saka *hvatuñ, cf. the attested Soghdian words *xwt'w* ‘ruler’ (< *hva-tāvya-) and *xwt'yn* ‘wife of the ruler’ (< *hva-tāvyani). For a possible Eastern Iranian etymology of another title, khagan, see Section 2.2.

Of these titles, only khagan was attested among the European Avars in the early period of their history. In general, the evidence on the language(s) of the Avars before the eighth century AD is extremely scarce. Almost all we have is a few personal names whose interpretation requires caution. The name that has attracted most attention from historical linguists is that of Bayan (*Βαιαν-ος*), the first khagan of the Avars (r. 562–602). It can be analyzed as *bayan ‘rich’ in Bulghar Turkic and Mongolic, the word known to be used as a personal name among both speaking communities. Etymologically, it is an element of Turkic origin in Mongolic, not *vice versa*, as shown by Dybo (2011: 132–133). On its own, this does not assure that the bearer of this name was a Bulghar Turkic speaker; it cannot be excluded that he was a Bulgharized Mongolic speaker or even a speaker of a third language, on condition that its naming system included this name of Bulghar Turkic origin. However, what is remarkable is that the same Bulghar Turkic (or, at least, broadly Turkic) origin can be rather safely assumed for at least some of the other Avar names from the early period (Bookolabras, Kokh, maybe also Solakhos and Samur). The early Avar names also included items of local Germanic (Gepidic) and Iranian (Alanic) origin (Pohl, 2019: 271), but no linguistic trace of Mongolic can be seen there.

Along with khagan, a few more titles are known from the late Avar period: *iugurrus*, *tudun*, *tarkhan*, *canizauci*. According to Pohl (*op. cit.*: 353–366), they must have been introduced into the Avar political system at some point after 626 AD. While the ultimate origin of these title terms is debatable, at least some of them can be reconstructed as Proto-Turkic terms (e.g. *tudun), and their spread across the Eurasian steppe zone is most likely associated with the early Turkic migrations. The closest parallels to these Avar title terms are found among the contemporary Bulghars and the Khazars.

**Figure 3.**
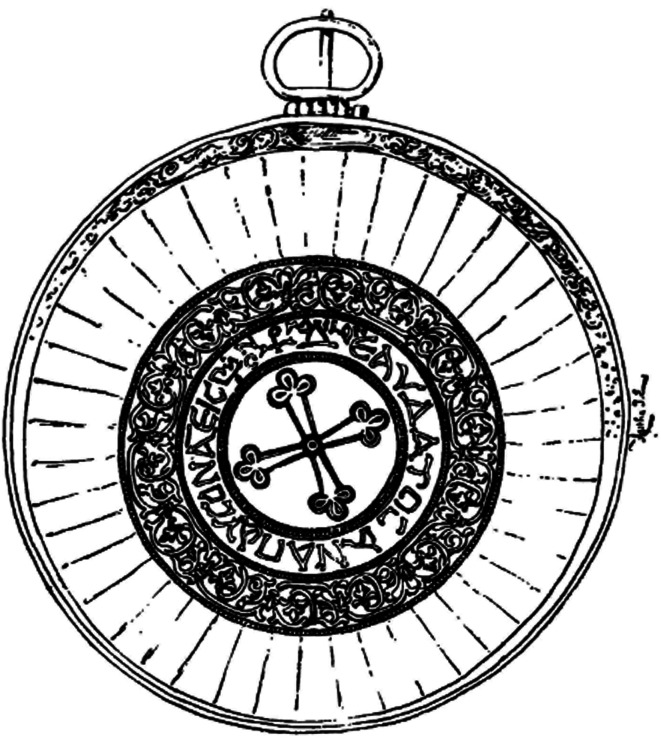
The Buila inscription from the Treasure of Nagyszentmiklós (reproduced from Hampel, 1894).

The most important textual piece of evidence on Avar is the so-called Buila inscription, a short record of an ‘unknown’ language placed on a golden bowl from the Treasure of Nagyszentmiklós (found in 1799 in the Hungarian Transylvania, now in western Romania). The text contains nine words written in Greek letters (Figure 3). Like the Treasure itself, the inscription is poorly datable. Based on the historical context, Róna-Tas and Berta (2011: 1163) assume that the Treasure was hidden after the defeat of the Avar Khaganate in the early ninth century AD.

Different readings of the text are available in the literature, but most authors agree that the inscription attests an early Turkic variety (Thomsen, 1918; Mladenov, 1927; Németh, 1932; Haussig, 1985; Erdal, 1988; Róna-Tas, 1990). In terms of historical Turkic phonology, the most up-to-date reading was provided by Mudrak (2005). He offers an analysis based on Bulghar Turkic material, involving both the scarce evidence from the extinct Bulghar varieties and the data of the contemporary Chuvash, the only surviving Bulghar Turkic language. Among the non-Turkic readings, the most ambitious one was that by Helimski (2000; 2003). He believed that the Buila inscription had been made in the early Avar period and argued for a Tungusic affiliation of Rouran and Early Avar, while accepting that the late Avars were Bulghar Turkic speakers. Recently, de la Fuente (2015) scrutinized thoroughly Helimski's Tungusic reading and concluded that is incompatible with the state of the art in Tungusic historical linguistics. That the Buila iscription was written in a Bulghar Turkic variety remains, thus, the most plausible hypothesis. A caveat is, however, that its identification with the Avar language is not quite secure as the bowl may, in fact, have come to the Avars as booty or present (Pohl, 2019: 367).

In parallel with Helimski, Futaky (2001) advocated the Tungusic–Avar hypothesis based on the alleged presence of Tungusic loans in the languages of the Carpathian basin. His proposal was refuted by Kara (2002) and then by Róna-Tas (2003). Yet another idea, suggesting a Mongolic affiliation of the European Avars (cf. Pelliot, 1915; Pulleyblank, 1962; Menges, 1979; Harmatta, 1983; Ligeti, 1986), was drawn mainly from the name of Bayan (see above) and the fact that some Mongolic loans had reached Hungarian and South Slavic. However, as shown by Róna-Tas and Berta (2011), these words should be seen as loans from Middle Mongolian through the mediation of the Cumans, who fled into the Carpathian basin after 1220. Regarding the more complicated case of South Slavic *xorǫgy ‘flag’, a Mongolic etymology (cf. Menges, 1979: 158) must be abandoned in favour of a plausible Bulghar Turkic source (Dybo, 2007: 48; Róna-Tas and Berta, 2011: 434).

The last type of evidence that may represent the language(s) of the Avars is two dozen very short inscriptions made in the Pannonian variant of the so-called Eastern European runic alphabet (Kyzlasov, 1994). There is no universally accepted reading of this script; for general historical reasons, its language is usually seen as Bulghar Turkic (e.g. Kyzlasov, 2012: 235). Mudrak (2017) has offered readings of Eastern European runic inscriptions based on Ossetic. This interpretation implies that some groups of Alanic origin were part of the population of the Avar Khaganate.

### 3.3. Population genetics

Genetic study of Avars and their possible progenitor groups, such as Rourans and Xianbei, is very rare, and most studies focused on uniparental markers as in the case of Xiongnu. Recent studies on mitochondrial and Y haplogroups of Avar elites report a substantial fraction of their haplogroups with broadly eastern Eurasian origin (Csáky et al., 2020; Neparáczki et al., 2019). The vast majority of their Y chromosomes with eastern Eurasian origin belong to haplogroup N with a few of Q and C, suggesting their northern Asian origin and possibly a rather homogenous eastern source population at least with regard to the paternal ancestry (Csáky et al., 2020; Neparáczki et al., 2019). The remaining haplogroups are of western Eurasian origin, implying admixture and heterogeneous origin of the Avar group, while it is beyond the resolution of uniparental markers to investigate if this genetic heterogeneity represents a socioethnic structure (e.g. some individuals with entirely eastern Eurasian ancestry and the others with entirely local western Eurasian ancestry) or an admixed population. Genomic study of ancient Avar elites is critical to understand the true nature of the Avar genetic profile.

**Figure 4.**
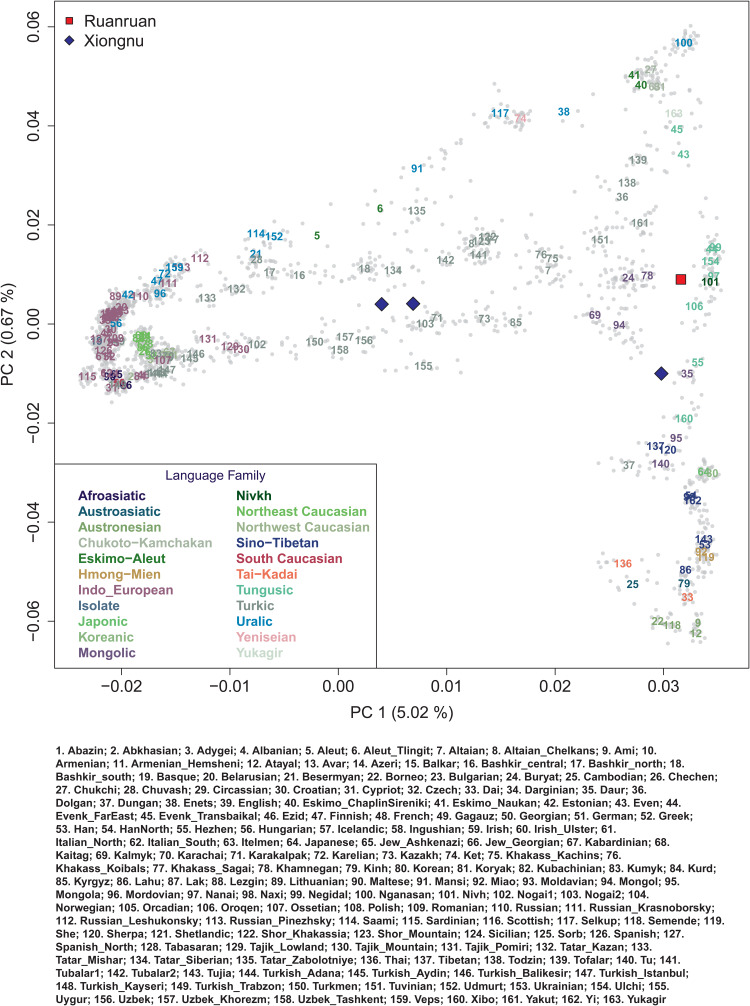
The genetic affinity of the Xiongnu- and Rouran-period individuals with present-day Eurasian populations. We present the first two principal components calculated for 2077 present-day Eurasian individuals and project the ancient individuals on top of it. The Rouran-period individual falls between present-day Mongolic-speaking populations from southern Siberia and Tungusic- and Nivkh-speaking ones from the lower Amur river basin. Each grey dot represents a present-day individual, and the coloured numbers represent the average coordinates of the populations, coloured by their language family. Population IDs corresponding to the numbers are provided at the bottom of the plot. Data are sourced from previous publications (Damgaard et al., 2018a; Jeong et al., 2019; Li et al., 2018).

A dearth of archaeogenomic resources from the eastern steppe during Xianbei and Rouran periods makes it even more difficult to characterize the Avar genetic profile. Until now, only a single Rouran-period genome has been published (Li et al., 2018). This individual has a genome-wide ancestry largely similar to present-day northern Asian, distinct from the three published Xiongnu genomes. In a descriptive principal component analysis, it falls between present-day Mongolic- and Tungusic-speaking populations from Mongolia and the lower Amur river basin, respectively (Figure 4). We speculate that this genetic profile matches well with the supposed geographic origin of Xianbei (and therefore Rourans) in the Greater Khingan mountains that separates Mongolia from Manchuria, while acknowledging that a single individual may not be a representative of the entire population. Until more genomic data become available from both sides, it will remain unclear whether the Avars were specifically related to Rourans, while limited data already suggest that both groups have an ancestry related to present-day north Asians, particularly the Altaic speakers (to the exclusion of the Uralic/Yeniseian ones).

## 4. Discussion and conclusion

The critical evaluation of the evidence available to individual disciplines, which we performed in Sections 2 and 3, offers a basis for interdisciplinary insights into both the Xiongnu/Hunnic and Rouran/Avar problems. Different lines of evidence provide support for a mixed eastern/western Eurasian origin of the Xiongnu of Inner Asia. As is suggested by archaeology and cultural history, the core Xiongnu population in eastern Mongolia may have included an Iranian (Saka) component or, at least, the Xiongnu were strongly affected by the Iranians. From a linguistic viewpoint, this component can be associated with the items of Eastern Iranian origin in the reconstructed part of the Xiongnu vocabulary. The predominant part of the Xiongnu population is likely to have spoken Turkic (Late Proto-Turkic, to be more precise). This picture seems to be mirrored in the genetic profile of the Xiongnu, suggesting a mixture of a western Eurasian ancestry (which is related to modern and ancient Iranian populations, among others) and an eastern Eurasian genetic substratum. Our linguistic analysis finds evidence for a Yeniseian affiliation of the Xiongnu, or a part of them, unconvincing; nor is the Yeniseian hypothesis supported by population genetics.

The evidence for a continuity between the Xiongnu of Inner Asia and the Huns of Europe is very weak, largely because of the overall scarcity of an eastern Eurasian component in the interdisciplinary profile of the Huns. The eastern steppe heritage is extremely limited in their archaeological record, and surprisingly no ancient genome from the Hunnic period Carpathian basin has been reported to test the eastern Eurasian genetic connection. The few common nouns that were recorded as part of the European Hunnic vocabulary are all of local origin, and the personal names of the Huns include items that are connected to the Indo-European languages of Europe (Germanic and Ossetic, in particular). This implies a crucial role of Western Eurasian components in the formation of the Huns. The titles of the Huns are broadly related to the steppe nomadic world, but no specific connection with the early Turkic speakers of eastern steppe (respectively the Xiongnu as their historical and archaeological counterpart) can be firmly established on this basis. The ambiguity of possible interpretations is as much the case for the Hunnic personal names for which a Turkic origin was previously proposed. To sum up, while historical and archaeological evidence may imply the inclusion of some steppe component among the Huns, the very limited linguistic and genetic data do not provide support for linking this component with the eastern part of the Eurasian steppe, or the Xiongnu specifically.

The interdisciplinary evidence on the Asian Rourans is even more limited. While Rouran archaeology is still in its infancy, the scarce genetic evidence suggests broadly Northeast Asian ancestry for the Rourans. Linguistic interpretation is largely hindered by the fact that none of the fragmentary materials that are discussed as the remnants of Rouran in the literature can be reliably associated with the main language of the Rouran population or its elite. The hypothesis on a Mongolic affiliation of the Rourans seems most ambitious to date, and if proved to be correct, this proposal would, at least, not contradict the genetic results.

The broadly East Asian component in the archaeological record of the European Avars is limited even in the earlier period of their history; elements originating from West Asia, the Caucasus, the Southern Russian steppes and the local Central European cultures can be traced alongside each other. From a linguistic perspective, there is a general consensus that the Late Avars were speakers of a Bulghar Turkic variety. The linguistic profile of the Early Avars is more controversial because of the scarcity of available evidence. Yet an identification of the Early Avars as Bulghar Turkic speakers looks much more plausible as compared with the alternative proposals, such as Mongolic or Tungusic. As long as there is no clear data identifying Rouran as a Bulghar Turkic language, and while the hypothesis on a Mongolic affiliation of the Rourans remains under consideration, the linguistic continuity between the two groups should be taken as an unproven allegation. Another option to discuss is a historical and cultural – but not linguistic – continuity; this would imply a language shift from the Mongolic-speaking Rourans to the Turkic-speaking Avars at some point of their history. There is, however, no proper linguistic evidence (e.g. demonstrable Mongolic substratum in the language of the European Avars) that would support this model. Therefore, yet another possibility should be considered, namely that, in accordance with Pohl's (2019) ‘Pseudo-Avar’ theory, the European Avars adopted the Rouran political identity but were of different origin and spoke a different language. Population genetics in the current state of research is neutral as regards the question of continuity between the Rourans and the Avars. What it is supported is that at least some European Avar individuals were of Eastern Asian ancestry, be it Rouran-related or not.

## Data Availability

All data used for this article can be found in the published literature cited in the references.
