# Early nomads of the Eastern Steppe and their tentative connections in the West – CORRIGENDUM

**DOI:** 10.1017/ehs.2022.8

**Published:** 2022-03-10

**Authors:** Alexander Savelyev, Choongwon Jeong

The authors apologise that within the above article, Savelyev, A., & Jeong, C. (2020), the image used for figure 3 was incorrect.

The correct image is as below.

**Figure 3.**
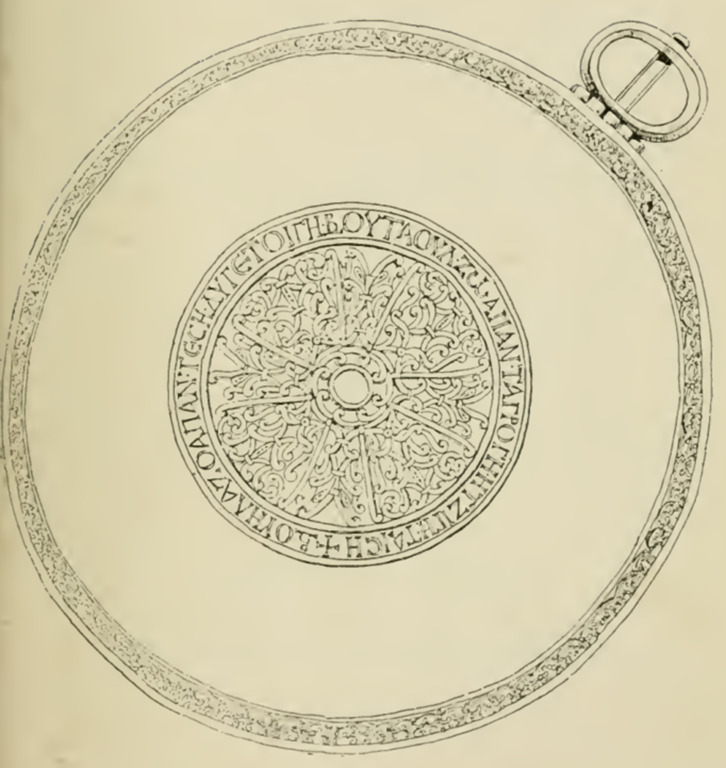
The Buila inscription from the Treasure of Nagyszentmiklós (reproduced from Hampel, 1894).

The authors would like thank Dr. Béla Kempf who drew their attention to this error.
